# A Time Series Proposal Model to Define the Speed of Carbon Steel Corrosion in an Extreme Acid Environment

**DOI:** 10.3390/ma18010027

**Published:** 2024-12-25

**Authors:** Juan Carlos Fortes, Ana Teresa Luís, María Santisteban, José Antonio Grande

**Affiliations:** 1 Sustainable Mining Engineering Research Group, Department of Mining, Mechanic, Energetic and Construction Engineering, Higher Technical School of Engineering, University of Huelva, 21007 Huelva, Spain; jcfortes@uhu.es (J.C.F.); maria.santisteban@dimme.uhu.es (M.S.); grangil@uhu.es (J.A.G.); 2 Department of Water, Mining and Environment, Scientific and Technological Center of Huelva, University of Huelva, 21007 Huelva, Spain

**Keywords:** acid mine drainage, corrosion steel, durability, time series analysis, predictive model

## Abstract

This article shows the behavior of the corrosive effect of acid mine water on carbon steel metal alloys. Mining equipment, composed of various steel alloys, is particularly prone to damage from highly acidic water. This corrosion results in material thinning, brittle fractures, fatigue cracks, and ultimately, equipment failure. For this purpose, a set of carbon steel metal plates similar to those found in mine facilities were immersed into mine leachates of an AMD (Acid Mine Drainage) polluted river from the Tharsis Mine (Huelva, Spain). In these leachates, physicochemical variations occur, directly correlated with the alterations produced in the metal plates, manifested with the appearance of dissolved materials and particulate matter. Weight loss of up to 37 g in 30 weeks for plates of about 140 grs occurred and an increase in EC up to 45.64 mS/cm from 5.40 mS/cm and an increase in TDS from 2600 mg/L to 17,100 mg/L. STATGRAPHICS Centurion, a powerful data analysis tool was used for performing the time series analysis that was used for the first time to statistically define the corrosion effects on metal alloys. As a result, a significant variability in the physical and chemical factors of the leachates was observed due to the redox and precipitation–dissolution processes occurring within the system: an increase in total dissolved solids (TDS), electrical conductivity (EC) and temperature (T) (the corrosion process is an exothermic reaction) and a decrease in pH. It was also demonstrated that the longer the exposure time, the plates noticeably lost more material and became further weakened. Finally, these results allowed the formulation of a simple algorithm to define weight loss as a function of exposure time to acidic water.

## 1. Introduction

The problem of AMD is that polymetallic sulfides mining, and, more specifically, iron sulfides, generates a very acidic (pH < 3) leachate with a high concentration of dissolved sulfates, metals, metalloids, and rare earth elements (REE) [1,2,3,4,5,6] denominated as Acid Mine Drainage (AMD). This is a global problem [7] not only due to the pollution it produces, directly affecting the water environment, but also due to the number of indirect problems it generates. AMD is not only associated with surface and groundwater deterioration but also with the degradation of soil quality, aquatic habitats, and assimilation of heavy metals in the environment [8]. While AMD can occur naturally, it is significantly exacerbated by sulfide mining operations, whether in open mines or in closed and abandoned facilities throughout the centuries. Examples of this can be observed in polymetallic sulfide mining operations and in a lesser extent in coal mines, and can be found in South America (Chile and Peru), Asia (Indonesia), North America (Canada and USA), Africa (Zambia) and Europe. In SW Europe, the Iberian Pyritic Belt (IPB) has a high concentration of sulfurous minerals and as a result of mining exploitation, a large amount of AMD is still present [9,10,11].

Environmental impacts: AMD results in the release of hydrogen ions and the marked decrease in pH which can even be negative [12], which makes it a prominent environmental problem without an effective solution nowadays.

Furthermore, although this process can be slow, the existence of ferric ion and the presence of a high number of chemolithotrophic bacteria (such as *Acidithiobacillus ferrooxidans y Leptopirillum ferrooxidans*) can make this process 51 to 100 times faster [13].

The basic reactions governing this process were described in detail by Younger et al. (2002) [14] in Equations (1) and (2) [15]:

(1) $$FeS_{2}+7/2O_{2}+H_{2}O\to Fe^{2+}+2{SO}_{4}^{2-}+2H^{+}$$

(2) $$Fe^{2+}+1/4O_{2}+H^{+}\to Fe^{3+}+1/{2H}_{2}O$$

These equations detail the oxidation process of sulfides under the influence of water, oxygen, and microorganisms [16,17,18,19,20,21].

Apart from the numerous environmental problems caused by AMD, water quality is the most important element affected as it undergoes physical and chemical changes that cause a sharp drop in pH values. However, there are other economic and security concerns. Machinery, both indoor (pumps, electric motors, screws, drilling machines, support structures, wagons, rails, etc.) and outdoors (excavators, sieves, crushers, conveyor belts, trucks, etc.) and constructions [22,23] that are exposed to these extremely harsh environmental conditions pose challenges in terms of durability, user safety, and high repair costs [24,25,26,27,28].

Metallic materials are damaged by corrosion processes that include chemical and electrochemical reactions that can be accelerated depending on the environment to which they are exposed [29,30] which becomes evident in particularly aggressive environments such as AMD [31,32,33]. This process results in loss of material and strength of metallic materials, reducing their performance and raising safety, stability, and economic concerns, highlighting the importance of process analysis.

Existing research: There are numerous scientific studies on the effects of salt water on metal alloys [34,35,36]. However, the impact on machinery components in mining environments affected by Acid Mine Drainage (AMD) is a relatively underexplored area in the scientific literature [37,38].

The objective of this paper is to evaluate the effects of AMD corrosion in materials manufactured with steel, which is an innovative topic, as well as analyze the data (Weight loss, EC, TDS, pH, and T, which are altered during the corrosion study) using descriptive statistical analysis methods, thus obtaining the parameters that distinguish the characteristics of a dataset.

## 2. Materials and Methods

### 2.1. Experimental Design

To carry out this experiment, a six-meter-long metal platen made of carbon steel with a carbon percentage of less than 0.25% was used, from which thirty metal plates of approximately 0.05 × 0.06 × 0.006 m were obtained (the width and thickness measurements were common to all, but the length varied due to the mechanical cut they received) and used for the experiment. Their chemical composition, given in % (material properties extracted from certification tests): C = 0.20; Mn = 0.49; Si = 0.21; S = 0.017; *p* = 0.011; Cu = 0.22; Cr = 0.08; Ni = 0.11; Ceq = 0.32; and ambient temp.

The water used for the experiment was sampled from an AMD affected river that receives polluted water from the Tharsis Mine [39]. This iconic watercourse is in The Iberian Pyrite Belt (IPB), one of the world’s largest sulfide-forming provinces, with a mining history of more than 5000 years without any preventive or corrective measures [40]. The water was taken to the laboratory in polypropylene containers previously cleaned with distilled water with 10% nitric acid concentration.

The initial physicochemical properties of the AMD affected water were the following: pH = 2.9, redox potential (Eh) = 220 mV, total dissolved solids (TDS) = 2.41 mg/L, and electrical conductivity (EC) = 4.9 mS/cm. A 50 L stock of the same water was maintained in an open recipient and stirred to replenish the reagent solution as it evaporates.

In the laboratory, the AMD affected water was put in 0.8L containers and was constantly moving with a stirrer. On the 30 January 2021, 30 plates were placed on a rounded plastic support to be elevated and immersed in the containers, ensuring maximum contact with the water. The submerged plates were taken every week (1 per week), washed and dried before being measured (weight, width, height, and thickness). The physicochemical parameters, pH, EC, TDS, Eh, and temperature (T), of the water were measured weekly, just before removing the plate with a HORIBA LAQUA PC-110-K multi-parameter meter.

The plates remained in contact with the solution for different durations, with the first plate being immersed for one week and the last plate for 30 weeks, until 9 June 2021. All throughout the experiment, the plates were kept still.

### 2.2. Statistical Treatment

The results obtained from the plates’ measurements as well as the physico-chemical data of the resulting water were compiled into an excel matrix for further graphical-statistical treatment. The Statgraphics Centurion XVI software (version 16.2.04, Statgraphics Technologies, Inc. (The Plains, VA, USA) was used for the data analysis [41,42,43,44]. It is a technique widely used in mining activity affected environments [45,46,47,48] and gives a set of features for exploratory data analysis, statistical summary, analysis of variance, statistical control, multivariate analysis, etc., allowing the classification of the different studied variables into categories or proximity ratios.

It is a powerful data analysis tool that combines a wide range of procedures with interactive graphs to provide an integrated data analysis environment. This software is designed to facilitate the statistical analysis of data and through its application it is possible to carry out a descriptive analysis of one or several variables, using graphs that explain their distribution or calculating their characteristic measurements. Among its many features are also the calculation of confidence intervals, hypothesis testing, regression analysis, multivariate analysis, as well as various techniques applied in Quality Control.

By definition, descriptive research studies a phenomenon under natural conditions without considering hypotheses, that means, this design does not allow causal hypotheses to be corroborated or falsified (only descriptive or exploration of associations), but rather generates them as a basis for analytical studies [49]. Descriptive statistics are concerned with presenting the most important information of a summarized dataset [50]. To do this, its central measurements are calculated (mean, median…) and a measure is given of how the data are dispersed around those central values (variance, standard deviation, range…). Likewise, after a descriptive analysis, a representation of the data will be available in the form of graphs, so that it is possible to detect outliers, trends, or groupings.

The time series procedure is designed to forecast future values of time series data. A time series consists of a group of sequential numerical data taken at equally spaced intervals, usually over a period of time or space. This procedure tests many models and selects one that has the best performance according to a specified criterion. The procedure graphs the data and can display the autocorrelations, partial autocorrelations, and the periodogram of the sample. Tests are performed to determine whether the observations could be samples of a random process or “white noise”. If a second time series is provided, cross-correlations between the two series are also calculated and displayed.

The Multiple Regression procedure is designed to construct a statistical model that describes the impact of two or more quantitative factors X in a dependent variable Y. The procedure includes an option to run a stepwise regression, in which a subgroup of X variables is selected. The fitted model can be used to make predictions, including confidence limits and/or prediction limits. Residuals can also be plotted and influential observations identified.

## 3. Results and Discussion

### 3.1. Statistical Summary

Table 1 shows the statistical summary of the physical-chemical data obtained from the different samples analyzed during the thirty weeks, the duration of the experiment, where it can be seen how the maximum exposure time was 219 days, while the minimum was 9 days, in which the maximum weight loss of the affected plates was 37.0 g and the minimum was 1.98 g, affecting a maximum volume and surface of 17.9 cm^3^ and 74.0 g, respectively, while the minimum values recorded for these statistics have been 14.1 cm^3^ and 66.9 g.

In the acid water, the recorded data show maximum temperature of 24.5 and minimum of 10.5. Maximum pH values of 3.60 and minimum values of 2.05, presenting a coefficient of variation of 15.6%. Regarding EC, the maximum value recorded was 45.64 mS/cm, while the minimum was 5.40 mS/cm. Thus, the TDS has a maximum of 17,100 mg/L and a minimum of 2600 mg/L. Both parameters present high values of the coefficient of variation, obtaining values of 63.1% for EC and 56.9% for TDS. The Eh has a maximum of 392 Mv and a minimum of 190 Mv and a coefficient of variation of 21.2%.

In the palates, the variation coefficient for surface area and volume was not very high (2.62% and 7.30%, respectively), being more significant for exposure time (56.8%) and for weight loss (73.7%).

These results are consistent with other studies carried out on this subject [27,48,51].

**Figure 1:**
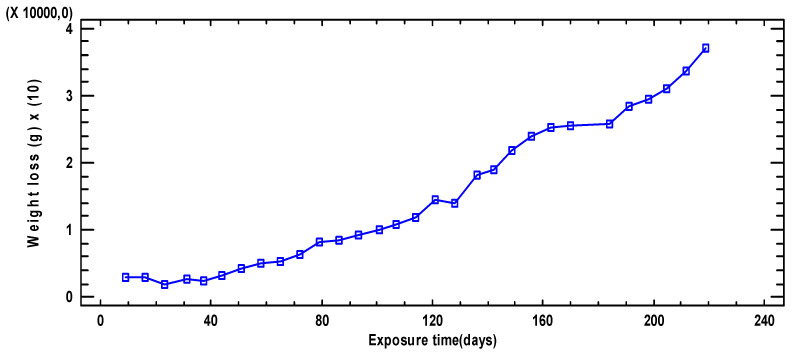
Evolution of weight loss with exposure time in the plates.

Figure 1 represents the evolution of weight loss versus exposure time. It is possible to see how as the exposure time increases and the weight loss increases, as expected.

### 3.2. Autocorrelation Function (ACF)

In time series analysis, the ACF indicates the correlation between a variable and itself, that is, the Pearson r that the variable takes on any given day with the r value of the previous, next, two following days, etc.

**Figure 2:**
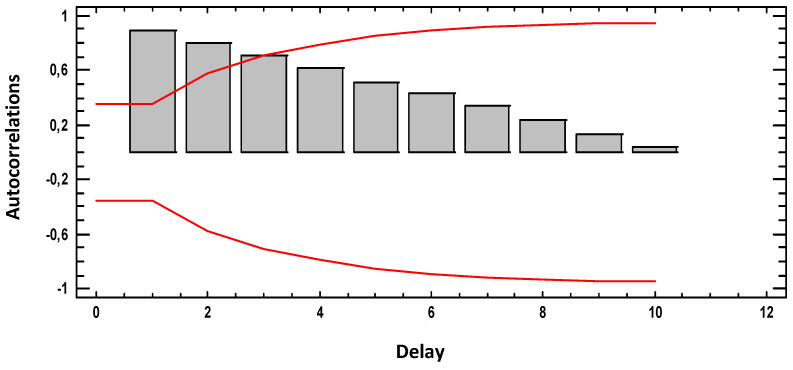
Estimated autocorrelations for weight loss (g).

Figure 2 shows the estimated autocorrelations between the weight loss (g) × (10) values at different lags. The autocorrelation coefficient with lag k measures the correlation between the values of weight loss (g) × (10) at time t and time t-k. Also shown are 95.0% probability bounds around 0. If the probability bounds at a particular lag do not contain the estimated coefficient, there is a statistically significant correlation at that lag at the 95.0% confidence level.

Remember that the concept LAG in statistics means time between two measurements or delay. As in the graph we take 30 measurements in 30 weeks, and as in the graph the 30 weeks occupy 10 LAG, then each LAG is equivalent to 3 weeks of time. The red line indicates that the values that are above it (Pearson’s r values (vertical bars)) or exceed the system memory. In our case, the graph shows that we have two and a half lags above, that is 7.5 weeks. Beyond those 7.5 weeks, the system does not remember.

### 3.3. Cross-Correlations Function

**Figure 3:**
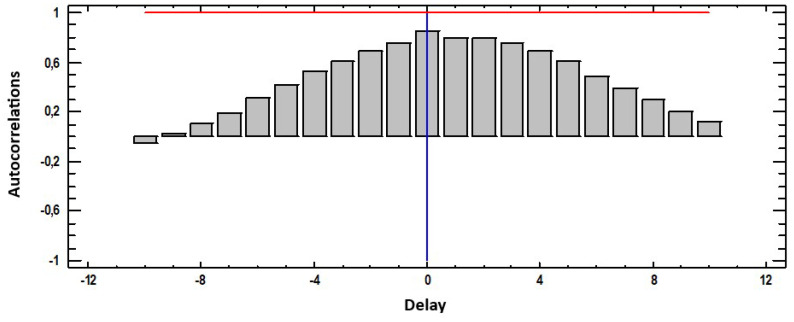
Cross-correlations weight loss (g) with temperature (°C).

**Figure 4:**
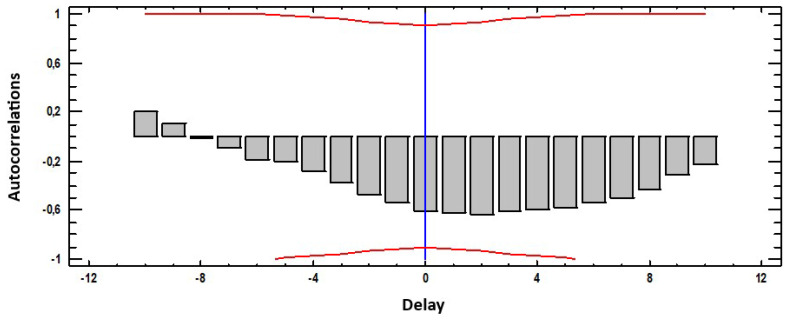
Cross-correlations weight loss with pH.

**Figure 5:**
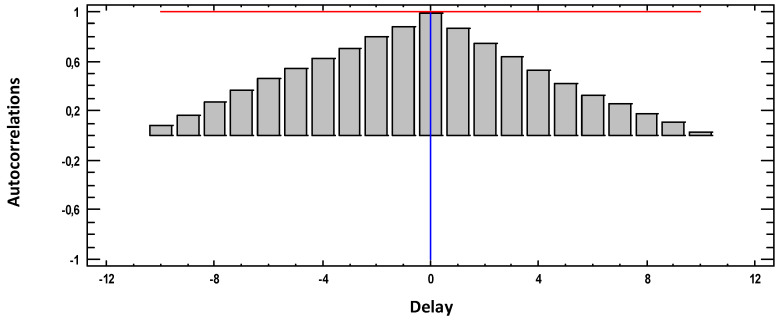
Cross-correlations weight loss with EC.

**Figure 6:**
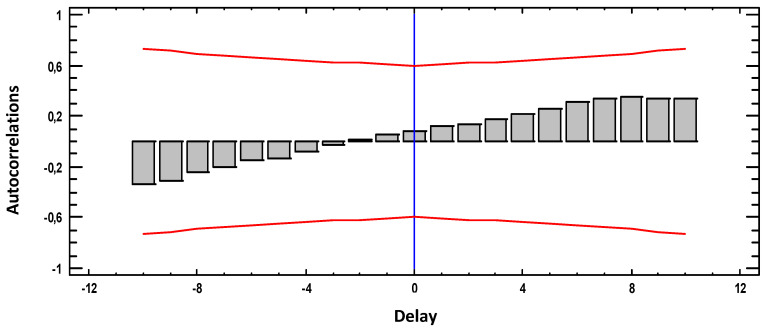
Cross-correlations weight loss with Eh.

**Figure 7:**
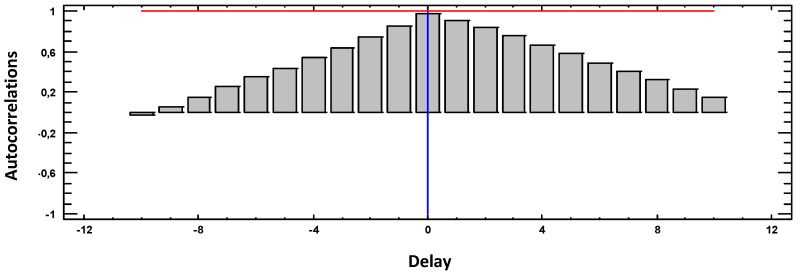
Cross-correlations weight loss with time exposure.

Figure 3, Figure 4, Figure 5, Figure 6 and Figure 7 present the results of the cross-correlation functions between pairs of variables for the 30 sampled weeks. Thus, each LAG represents 3 weeks of sampling.

Figure 3 shows the cross-correlation between weight loss and temperature, showing a high correlation between both variables with a maximum of 0.9 at T = 0, which implies that increases or decreases in the values of some of the parameters occur practically simultaneously and positively, that is, if the temperature increases, weight loss also increases almost at the same time.

Figure 4 shows the cross-correlation between weight loss and pH. In this case, the correlation is negative and somehow less significant than the previous case since it presents a maximum correlation of 0.6 at T = 2. Thus, decreases in pH values are translated into increases in weight loss values, and vice versa, 6 weeks later.

Figure 5 shows the cross-correlation between weight loss and electrical conductivity (CE). In this case, the correlation between variables is maximum, presenting values of 1, and positively at t = 0. In this way, increases in CE imply increases in weight loss, and vice versa, almost simultaneously.

Figure 6 shows the correlation between weight loss and Eh. In this case, a very insignificant negative correlation is observed, with maximum values of 0.3 at t = 10, that is, at 30 days. This means that increases in the values of one of the variables have an impact on the decrease in the values of the other 30 weeks later.

Figure 7 shows the cross-correlation between weight loss and exposure time, presenting a high positive correlation (0.9) at t = 0 so that increases or decreases in the values of the variables occur in the same way and of almost immediately between the variables.

**Figure 8:**
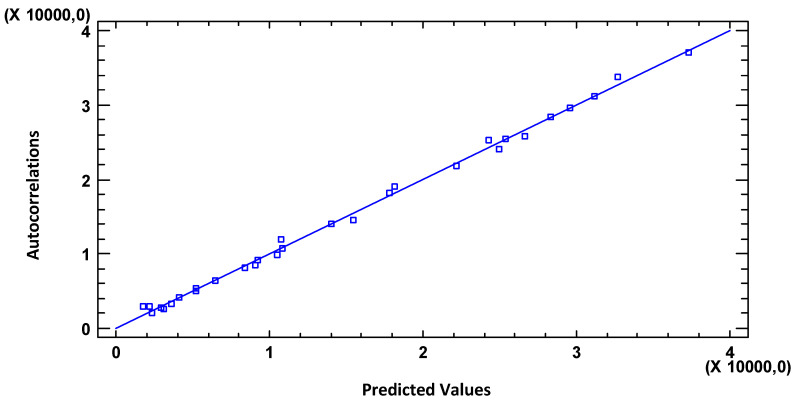
Weight loss graph with predicted values.

Figure 8 shows the results of fitting a multiple linear regression model to describe the relationship between weight loss (g) × (10) and eight independent variables. The equation of the fitted model is as follows: *weight loss (g) × (10) = 35263.2 + 34.2595∗exposure time (days) + 2.06251∗residual samples surface (cm^2^) − 0.515359∗residual samples volume × 20 (cm^3^)*.

Since the *p*-value in the ANOVA table is less than 0.05, there is a statistically significant relationship between the variables at a 95.0% confidence level.

The R-Square statistic indicates that the adjusted model explains 99.715% of the variability in weight loss (g) × (10). The adjusted R-Square statistic, which is most appropriate for comparing models with different numbers of independent variables, is 99.6821%. The Durbin–Watson (DW) statistic examines the residuals to determine if there is any significant correlation based on the order in which they are presented in the data file.

To determine if the model can be simplified, note that the highest *p*-value of the independent variables is 0.0001, which corresponds to exposure time (days). Since the *p*-value is less than 0.05, that term is statistically significant at the 95.0% confidence level. Consequently, it would not be good to eliminate any variables from the model.

**Figure 9:**
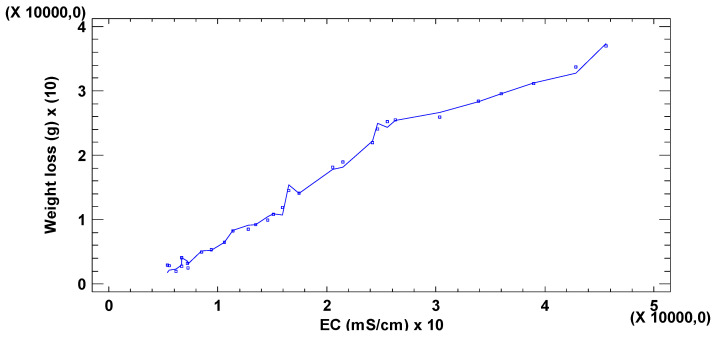
Weight loss graph with EC.

The graph of Figure 9 shows how the EC and the weight lost fit a linear function with a positive and discrete abscissa at the origin. In fact, this is to be expected as when the plates are attacked by the acidic water, they dissolve in it, thus the lost weight is transformed into dissolved matter (TDS), which in turn causes an increase in the EC of the immersion water.

The positive value of the abscissa (EC) for the zero ordinate (weight loss) is justified by the existence of an initial electrical conductivity prior to the introduction of the metal plates and characteristic of AMD media induced fundamentally by the ions present in the aqueous medium.

Corrosion is a well-researched issue, yet it continues to be challenging and costly. This study highlights the necessity to further investigate corrosion in mechanical and structural components exposed to AMD. It aims to analyze the chemical and electrochemical processes involved and identify the factors that significantly accelerate the degradation of these materials.

## 4. Conclusions

The main objective of this study is to study the effect of acid water in the corrosion process of carbon steel using time series tools, which will allow classic statistical conclusions to be demonstrated. For the first time, the use of time series to study corrosion processes in steel was carried out, which has proven to be an effective technique. This allows us to show the results numerically and also to quantify how acidic water affects the corrosion of the materials used in mining facilities, both those found in mining equipment and in the structural elements of mine facilities, and to define causal relationships.

The influence between the physicochemical fluctuations of the water and the deterioration due to weight loss of the metal plate immersed in it has been demonstrated. It can be concluded that acidic water had a great effect on carbon steel, causing a decrease in its volume and weight. It was observed how this corrosion process of steel plates causes changes in the physicochemistry of the water, provoking a significant increase in TDS and conductivity and a decrease in pH due to redox and precipitation–dissolution processes that occur within the system. The increase in temperature during the experiment is due to the corrosion process, which is an exothermic reaction.

On the other hand, the plate volume was expected to decrease due to the material loss caused by the oxidation–corrosion process. Naturally, exposure time is a critical factor in many experiments, especially those involving chemical reactions or environmental processes like acid mine drainage. The duration of exposure significantly influences the outcomes.

In summary, mining assets, including both machinery and structural components primarily made of steel and concrete, experience significantly accelerated corrosion in extremely acidic environments. This process is further intensified by acidophilic bacteria, which act as catalysts.

These results allowed the formulation of a simple algorithm to define weight loss as a function of exposure time to acidic water.

## Figures and Tables

**Table 1:** Statistic summary (count, mean, coefficient of variation (%), minimum, maximum) of the acid water variables T, pH, EC, TDS, Eh, and surface, volume, and weight of the plates, measured weekly during 30 weeks.

	T (Celsius)	pH	EC (mS/cm)	TDS (mg/L)	Eh (mV)	Exposure Time (Days)	Surface (cm^2^)	Volume (cm^3^)	Weight Loss (g)
**Count**	30	30	30	30	30	30	30	30	30
**Mean**	18.37	2.63	18.07	7564.35	258.32	112	71.08	16.58	14.79
**Coefficient of variation (%)**	22.7	15.6	63.1	56.9	21.2	56.8	2.62	7.30	73.7
**Minimum**	10.5	2.05	5.40	2600	190	9	66.9	14.1	1.98
**Maximum**	24.5	3.60	45.646	17,100	392	219	74.0	17.9	37.0

## Data Availability

Data is contained within the article.
